# Prediction of Pig Trade Movements in Different European Production Systems Using Exponential Random Graph Models

**DOI:** 10.3389/fvets.2017.00027

**Published:** 2017-03-03

**Authors:** Anne Relun, Vladimir Grosbois, Tsviatko Alexandrov, Jose M. Sánchez-Vizcaíno, Agnes Waret-Szkuta, Sophie Molia, Eric Marcel Charles Etter, Beatriz Martínez-López

**Affiliations:** ^1^Center for Animal Disease Modeling and Surveillance (CADMS), VM: Medicine and Epidemiology, University of California Davis, Davis, CA, USA; ^2^CIRAD, UPR AGIRs, Montpellier, France; ^3^Animal Health and Welfare Directorate, Bulgarian Food Safety Agency, Sofia, Bulgaria; ^4^Animal Health Center (VISAVET), Animal Health Department, Veterinary School, Complutense University of Madrid, Madrid, Spain; ^5^INRA, INP, ENVT, UMR 1225, IHAP, Université de Toulouse, Toulouse, France

**Keywords:** ERGM, network modeling, livestock contact networks, risk-based surveillance, infectious diseases

## Abstract

In most European countries, data regarding movements of live animals are routinely collected and can greatly aid predictive epidemic modeling. However, the use of complete movements’ dataset to conduct policy-relevant predictions has been so far limited by the massive amount of data that have to be processed (e.g., in intensive commercial systems) or the restricted availability of timely and updated records on animal movements (e.g., in areas where small-scale or extensive production is predominant). The aim of this study was to use exponential random graph models (ERGMs) to reproduce, understand, and predict pig trade networks in different European production systems. Three trade networks were built by aggregating movements of pig batches among premises (farms and trade operators) over 2011 in Bulgaria, Extremadura (Spain), and Côtes-d’Armor (France), where small-scale, extensive, and intensive pig production are predominant, respectively. Three ERGMs were fitted to each network with various demographic and geographic attributes of the nodes as well as six internal network configurations. Several statistical and graphical diagnostic methods were applied to assess the goodness of fit of the models. For all systems, both exogenous (attribute-based) and endogenous (network-based) processes appeared to govern the structure of pig trade network, and neither alone were capable of capturing all aspects of the network structure. Geographic mixing patterns strongly structured pig trade organization in the small-scale production system, whereas belonging to the same company or keeping pigs in the same housing system appeared to be key drivers of pig trade, in intensive and extensive production systems, respectively. Heterogeneous mixing between types of production also explained a part of network structure, whichever production system considered. Limited information is thus needed to capture most of the global structure of pig trade networks. Such findings will be useful to simplify trade networks analysis and better inform European policy makers on risk-based and more cost-effective prevention and control against swine diseases such as African swine fever, classical swine fever, or porcine reproductive and respiratory syndrome.

## Introduction

Movements of animals play a key role in the spread of several major infectious diseases, like foot-and-mouth disease, classical swine fever, or African swine fever (1–3). Therefore, detailed data on livestock movements may help to better simulate transmission dynamics and identify areas, periods, and farms that are more likely to spread the diseases and could be targeted to improve surveillance and control strategies (4, 5). However, one of the challenges of using livestock movement data to support decision-making in preventive veterinary medicine is the limited availability of timely and updated records on animal movements and, if available, the massive amount of data that have to be processed. This is particularly challenging when considering diverse and, sometimes, epidemiologically complex, production systems, such as backyard or extensive systems, where the information may not be frequently collected and accessible. Models of livestock movement networks based on holding characteristics and past-temporal observed networks could be useful to simplify real-world networks and to predict disease spread even in backyard or extensive environments.

Pig trade movements can be represented as a network, consisting of a set of nodes (here the pig premises) connected by links (also called edges) representing movements of pigs between them. These networks are not strictly identical from 1 year to the following, but their structural properties, which impact disease dynamics, are likely to be stable over time (6, 7). These properties emerge from pig trading behaviors. For example, some premises may be more likely to trade with each other due to geographical proximity or because they belong to the same pig company [selective mixing or homophily, see Morris (8)]. Some particular types of premises may also be more likely to trade with a high number of premises (attributes that influence degree). Finally, if a trading partner B of premises A trades with a third premises C, this might encourage A to trade with C (structural balance effect).

The first statistical models developed to evaluate which processes lead to observed network structures were quite simple. They only addressed relational reciprocation [i.e., mutuality; see Holland and Leinhardt (9)] or assortative mixing (8). The recent developments of exponential random graph models (ERGMs), also known as p* models (10), offer possibilities to better capture the complexity of real-life networks (11). This family of models assumes that the observed network is only one realization among many potential networks with similar characteristics and that the probability that a link exists is a logit-linear function of predictors that reflect node characteristics, link characteristics, and network structural properties (10, 12, 13). Although they were developed to handle the inherent non-independence of network data, the results of ERGMs are interpreted in similar ways to logistic regression, making this a very useful method for examining contact networks in the context of epidemiology.

The aims of this paper were to use ERGMs to (1) develop models that reproduce observed pig trade networks; (2) understand the mechanisms that underlie the organization of pig trade networks; and (3) predict trade networks structures in three different European pig production systems (i.e., industrial, extensive, and backyard). Results of this study are intended to inform the design of prevention and control programs for swine diseases such as African swine fever, classical swine fever, or porcine reproductive and respiratory syndrome under diverse epidemiological scenarios and pig productions systems in Europe.

## Materials and Methods

### Data Collection and Network Construction

Three areas were selected to represent different European pig production systems: Bulgaria, where most premises raise pigs for own consumption; the autonomous community of “Extremadura,” which is the cradle of extensive Iberian pig production in Spain; and the department of “Côtes-d’Armor,” which is the French department with the highest concentration of industrial pig premises.

Data on pig movements and premises characteristics were obtained from national databases, through Bulgarian Food Safety Agency in Bulgaria, the professional database of swine (La Base de Données Professionnelle Porcine—BDPORC) in France, and the Ministry of Agriculture, Food and Environment (MAGRAMA) in Spain. The year 2011, which was common in all databases, was retained for analysis. The premises characteristics available were the classification or type of farm (described in the next two sentences), the size of premises (i.e., number of sows, weaners, and finishers on farm), the type of housing system (i.e., indoor or outdoor), the geographical coordinates, and the pig company number (only for France). In Bulgaria, pig farms were classified as small producers (<10 pigs kept for own consumption), type B (medium-size: 10–500 pigs; with low biosecurity level: access to other pigs or feral pigs, use of swill feeding, no fences around the holdings, and/or no disinfection at the entrance and exit of buildings), type A (medium-size, high biosecurity level), or industrial farms (large size: >500 pigs; high biosecurity level) (14, 15). Traditionally and outdoor-raised East Balkan pig herds are also found in the South East of Bulgaria. For Spain and France, pig farms were classified as multipliers (premises that produce breeding stocks and semen), farrowing farms, farrow-to-finish farms, finishing farms, or small producers. Small producers for Spain were defined as those that produce pigs for own consumption, whereas for France were those with ≤4 pigs. Traders, collection centers, markets, fairs, and stopping points (i.e., or staging point: locations used to feed, water, rest, accommodate, care for, and dispatch animals in transit before arriving to their final destinations) were considered as trade operators. Because of the dead-end characteristics of slaughterhouses, these premises were excluded from analysis.

For each area, yearly networks (i.e., using year 2011) were built, the nodes being all pig premises of the study areas, even those that were not trading pigs during the study period. Movement data were aggregated over the study period, and a direct link was drawn whenever a shipment of pigs occurred between the corresponding premises. Movement imported from or exported to outside areas was excluded from the analyses.

### The ERGMs

Exponential random graph models specify the probability of any random network **Y** given a set of *n* nodes and their attributes as in Eq. 1.

(1) $$P_{\theta }\left(Y=y|n\;\text{nodes}\right)=\left(\frac{1}{c}\right)\exp\left(\sum_{k\text{=1}}^{\text{K}}\theta_{k}z_{k}(y)\right)$$

The *z_k_*(*y*) terms represent model covariates, any set of *K* network statistics calculated on the *y* observed network and hypothesized to affect the probability of this network forming. The model covariates can include network parameters that account for the frequency of occurrence of certain network configurations (e.g., two-path, triangles), as well as node or edgewise covariates like the pig company to which a premise belongs or the distance between two premises, respectively. The θ coefficients estimate the strength of the effect of each covariate. The denominator *c* represents the normalizing constant, which correspond to the sum of $\exp\left(\sum_{k\text{=1}}^{\text{K}}\theta_{k}z_{k}(y)\right)$ over all possible networks with *n* nodes.

Because ERGMs’ calculation time dramatically increased with the increase of network size, it was decided to exclude isolates, i.e., pig premises that did not trade with other premises, from the small-scale productions system (Bulgaria, initially 28,729 premises, of which 95.3% were isolated premises).

### Model Specification

First, an exploration of network data was undertaken, with the computation of several local topological measures (number of isolates, triangles, degree distribution, etc.) and of mixing matrices for premises’ attributes (16). Specifically, we computed the number of nodes, the network density, the percentage of isolates, the clustering coefficient, and the mean and range of in-degree and out-degree centrality measures [e.g., Ref. (5)]. Network graphs were plotted, with the nodes colored according to nodes’ attributes, to better visualize the selective mixing patterns.

Based on this exploration, several network statistics were chosen to represent hypothetic rules for trade movements (Table 1). *L*(*y*) captures the density of the observed network *y*. *M_i,v,a_*(*y*), *M_o,v,a_*(*y*), *H_a,v_*(*y*), *U_a,v_*(*y*), *S_a,v_*(*y*), and *E*(*y*) are attribute-specific terms that capture the way in which premise attributes structure trading patterns, where *a* represents the attribute (e.g., housing system) and *v* the level (e.g., indoor, outdoor). The main effects, *M_i,v,a_*(*y*) and *M_o,v,a_*(*y*), allow variation in the propensity of a premise to form incoming and outgoing edges according to the level of an attribute characterizing this premise. *H_a,v_*(*y*) models a tendency of edges to occur between premises belonging to the same attribute level that varies among attribute levels (hereafter referred to as *differential homophily*), while *U_a,v_*(*y*) models a uniform tendency of edges to occur between premises belonging to the same attribute level (hereafter referred to as *uniform homophily*). *S_a,v_*(*y*) accounts for variation in the occurrence of edges according to the levels of an attribute characterizing each of two premises (hereafter referred to as *selective mixing*). *E*(*y*) captures variation in the propensity of premises to form links according to the Euclidean distance in km to other premises.

**Table 1:** **Network statistics used to fit the exponential random graph models of pig trade networks**.

Network statistics *z_k_*(*y*)	Abbreviation used^a^
# of edges	*L*(*y*)
# of in- and outgoing edges for each type of production, housing system, pig company	*M_i,v,a_*(*y*), *M_o,v,a_*(*y*)
# of edges that are within housing systems, within pig companies, within regions, with differential homophily	*H_a,v_*(*y*)
# of edges that are within housing systems, within pig companies, within regions, with uniform homophily	*U_a,v_*(*y*)
# of edges that are within and between housing systems, within and between type of productions, within and between regions	*S_a,v_*(*y*)
Euclidean distance between pairs of premises	*E*(*y*)
# of isolates	*I*(*y*)
# of asymmetric links	*A*(*y*)
Geometrically weighted dyadwise shared partners	*gwdsp*(*y*, α)
Geometrically weighted edgewise shared partners	*gwesp*(*y*, α)
Geometrically weighted in- and out-degree distribution	*gwid*(*y*, α), *gwod*(*y*, α)

*^a^Some statistics use attribute-specific terms where a and v represent the attribute and level, respectively. The observed network is represented by y and the scale parameter by α*.

*A*(*y*) and *I*(*y*) model the tendency of premises to form unidirectional links or no links, respectively. The terms *gwdsp*(*y*, α), *gwesp*(*y*, α), *gwid*(*y*, α), and *gwod*(*y*, α) are related to local structures and represent the parametric forms of the alternating two-paths, clustering (alternating *k*-triangles) and in- and out-degree distributions, respectively. A fixed value of 0.5 was adopted for the scale parameter α in these terms (11).

The Markov Chain Monte Carlo (MCMC) algorithm was used to estimate the maximum likelihood for the θ coefficients included in models (12). The MCMC chain is intended to step around the sample space of possible networks, selecting a network at regular intervals to evaluate the statistics in the model. For each MCMC step, *n* (*n* = 1 in the simple case) toggles are proposed to change the dyad(s) to the opposite value. A chain burn-in of 10^5^ toggles, an MCMC sample size of 10^4^, and an interval between successive samples of 10^3^ was fixed for these models.

### Model Selection and Goodness of Fit

For each area, four models were built: (1) a simple Bernoulli model that only includes the number of edges; (2) a model with edges and statistics based on nodal attributes (hereafter called “edge + attribute” model); (3) a model with edges and structure-related statistics (“edge + network statistics” model); and (4) a model with edges, nodal attributes, and structure-related statistics (“edge + attributes + network statistics” model).

For the “edge + attribute” and “edge + network statistics” models, univariable analyses were performed first. The terms (i.e., attributes and network statistics) were then added one by one, until the best model fit was obtained. The fourth model was based on the final “edge + attribute” model, and network statistics terms were added one by one manually until the best model fit was obtained.

Three approaches were used to examine goodness of fit of the models: (1) check for model convergence and degeneracy; (2) comparison of Akaike information criteria; and (3) comparison of goodness of fit plotting for higher order statistics (11). For this purpose, four sets of statistics were used: the in- and out-degree distributions, the geodesic distance distribution, and the edgewise shared partner distribution, which reflects the clustering of the network (17). These statistics were chosen because of their impact on disease spread dynamics (18). Finally, plots of simulated networks were visually compared to the plot of the observed networks.

All analyses were conducted in R (19) using the “statnet” suite of packages (20, 21).

## Results

A total of 7,811 out of the 45,224 premises keeping pigs (i.e., 17.3%) were actively moving pigs during 2011. Description of the pig industry demographics (i.e., number of premises for each type of farm), pig trade (to/from different types of farm), and topological characteristics in Côtes-d’Armor (France), Bulgaria, and Extremadura (Spain) in 2011 are presented in Tables 2–4.

**Table 2:** **Description of pig industry in Bulgaria, Côtes-d’Armor (France), and Extremadura (Spain) in 2011**.

	Côtes-d’Armor	Extremadura		Bulgaria
Area (km^2^)	6,878	41,634	Area (km^2^)	110,944
Road density (km/km^2^)	2.9	0.21	Road density (km/km^2^)	0.36
# of premises	2,396	14,099	# of premises	28,729
Premise type (%)			Premise type (%)	
Multiplier	2.6	0.3	Multiplier	NA
Farrowing	2.9	1.9	Industrial^a^	0.21
Farrow-to-finish	45.4	65.8	Type A^a^	0.48
Finishing	47.5	28.6	Type B^a^	6.44
Small producer^b^	0.5	3.3	Small producer^b^	92.54
Unknown	1	0	East Balkan pigs^a^	0.33
Trade operator	0.1	0.01	Trade operator	NA
Outdoor premises (%)	1.6	38.9	Outdoor premises (%)	NA

*^a^For Bulgaria only: industrial farm (large size: >500 pigs, high biosecurity level farm); type A farm (medium-size: 10–500 pigs, high biosecurity level); type B farm (medium-size: 10–500 pigs, low biosecurity level); east Balkan pigs (traditional outdoor pig herds). Small producer for Bulgaria: <10 pigs kept for own consumption*.

*^b^Small producers were defined for Spain as those farms (any size) that produce pigs for own consumption; for France were those with ≤4 pigs and for Bulgaria were those with <10 pigs kept for own consumption. NA, not applicable/not available*.

**Table 3:** **Descriptive statistics of pig shipments in Bulgaria, Côtes-d’Armor (France), and Extremadura (Spain) in 2011**.

Country	# active premises^a^ (%)	# ingoing shipments per active premise		# outgoing shipments per active premise		Euclidean shipment distance (km)		Shipment size (# of pigs)	
		Median (IQR)	Max	Median (IQR)	Max	Median (IQR)	Max	Median (IQR)	Max
Bulgaria	1,349 (4.5)	1 (1–1)	121	3 (1–7)	107	3 (1–32)	433	4 (2–21)	1,750
Côtes-d’Armor	1,907 (79.6)	5 (2–9)	1,021	6 (3–13)	253	17 (5–34)	129	61 (6–207)	950
Extremadura	4,555 (32.3)	1 (1–1)	71	1 (1–2)	27	13 (1–35)	204	30 (7–103)	11,650

*IQR, interquartile range*.

*^a^Premises that sent or received pigs in 2011*.

**Table 4:** **Topological statistics of the pig trade networks in 2011**.

Production system (area)	Topological statistics					
	# of nodes	Density	% of isolates	Clustering coefficient	Mean *k*_in_ (range)	Mean *k*_out_ (range)
Small producers (Bulgaria)^a^	1,349	6.7 × 10^−4^	0.0	0.049	0.9 (0–7)	0.9 (0–35)
Extensive (Spain—Extremadura)	14,097	2.1 × 10^−5^	67.7	0.038	0.3 (0–70)	0.3 (0–27)
Intensive (France—Côtes-d’Armor)	2,396	5.4 × 10^−4^	20.4	0.066	1.3 (0–236)	1.3 (0–83)

*k_in_, in-degree; k_out_, out-degree*.

*^a^Isolates were excluded for Bulgaria: initially there were 28,729 premises, of which 95.3% were isolated premises*.

**Figure 1:**
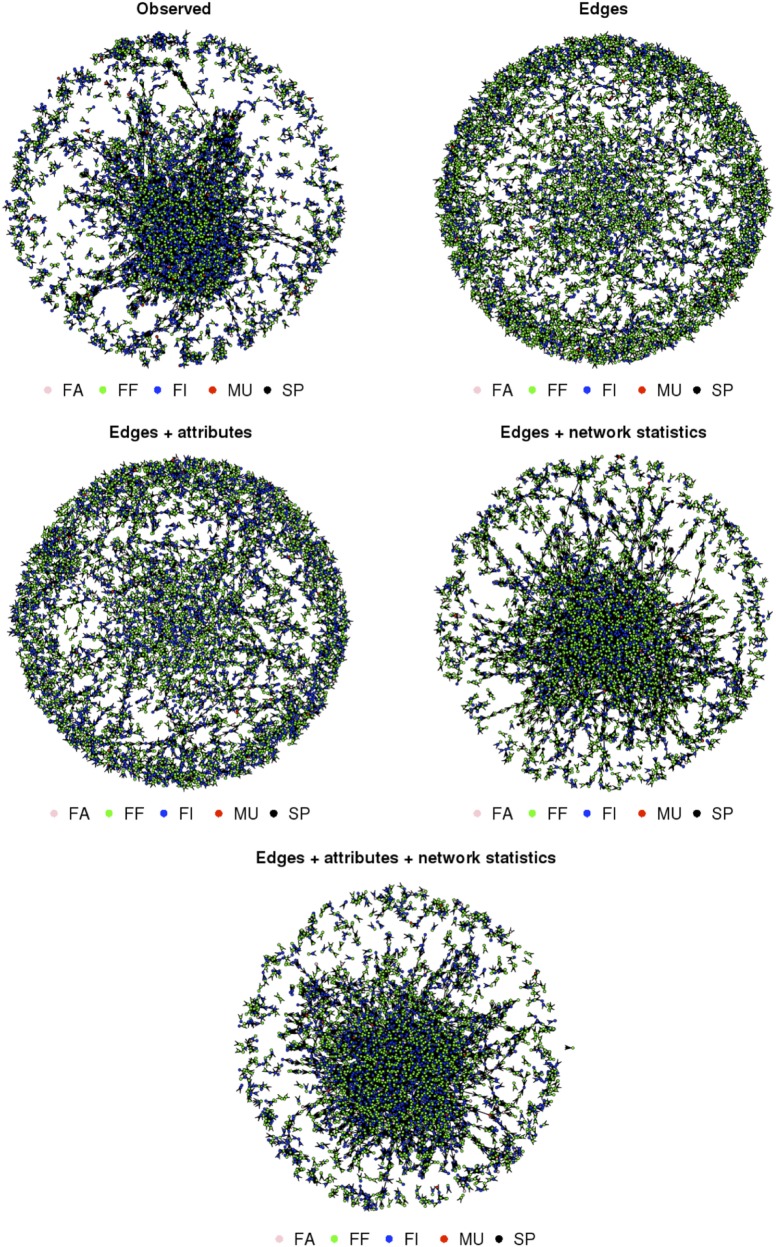
**Observed and simulated trade networks based on the four exponential random graph models in an extensive pig production system (Spain—Extremadura—2011); nodes colored according to their type of production: MU, multipliers; FA, farrowers; FF, farrow-to-finishers; FI, finishers; SP, small producers**.

**Figure 2:**
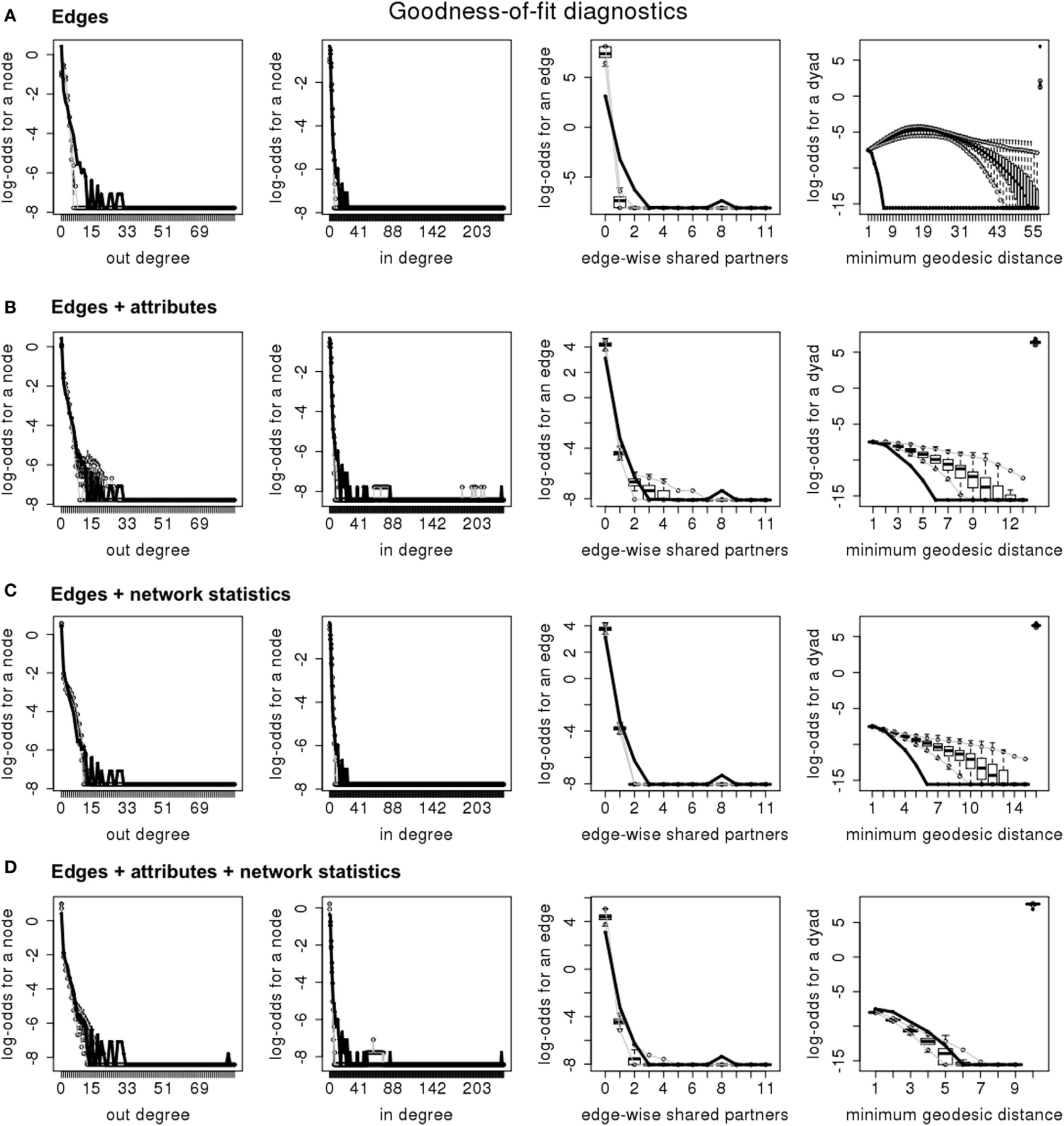
**Goodness of fit diagnosis for the four exponential random graph models in the intensive production system (France—2011); (A) “edges” model; (B) “edges + attributes” model; (C) “edges + network statistics” model; (D) “edges + attributes + network statistics” model**.

The inclusion of both nodal attributes and network configurations statistics provided the best fit to the data (Tables 5–7; Figures 1 and 2). Selective mixing between premises according to their type of production appeared to be an important mechanism of pig organization, whichever system considered (Tables 5–7). In addition to this mechanism, the other mechanisms related to premises characteristics that impacted the most on trade organization were belonging to the same pig company, the tendency of outdoors premise to trade with outdoor premises, and the geographical location of pig premises, in the intensive, extensive, and small-scale production systems, respectively (Tables 5–7). Network statistics on dyadwise and edgewise shared partner distributions, as well as on in- or out-degree distributions were needed to fit the models (Tables 5–7). These statistics better reflected the clustering of the observed networks and allowed to reproduce well the observed global network properties (Figures 1 and 2). This can be clearly observed in the goodness of fit diagnostics plot (Figure 2), where the value of all the observed network statistics (solid black line) is only well captured by the distribution of values of the simulated networks (underlying boxplots) generated with the final ERGM model (i.e., model with edges + attributes + network statistics).

**Table 5:** **Parameter coefficients and fit for the four exponential random graph models (ERGMs) of pig trade in a small-scale production system (Bulgaria)**.

Covariates	ERGM coefficients (SE)^a^			
	Bernoulli (edges)	Edges + attributes	Edges + network statistics	Edges + attributes + network statistics
*Edges*	−7.30 (0.03)***		−10.18 (0.30)***	−9.62 (0.67)***
*Link attributes*				
Distance (km)		−0.07 (0.00)***		−0.07 (0.00)***
*Nodal attribute mixing*				
Region^b^				
E to E		0.02 (0.16)		−0.88 (0.53)
NW to E		0.08 (0.48)		−0.36 (1.02)
S to E		0.92 (1.02)		1.85 (1.60)
SW to E		NA		NA
E to NW		0.57 (0.38)		−0.51 (0.74)
NW to NW		−1.29 (0.13)***		−0.82 (0.44)
S to NW		NA		NA
SW to NW		−1.43 (1.01)		3.10 (1.66)
E to S		3.50 (0.32)***		3.89 (0.68)***
NW to S		1.61 (0.37)***		1.75 (0.62)**
S to S		Reference		Reference
SW to S		NA		NA
E to SW		15.91 (0.49)***		20.78 (0.99)***
NW to SW		2.55 (0.31)***		6.49 (0.68)***
S to SW		3.77 (0.45)***		5.68 (1.06)***
SW to SW		0.39 (0.36)		1.75 (0.93)
Type of farm^c^				
SP to SP		Reference		Reference
IN to SP		2.37 (0.17)***		0.75 (0.24)**
TA to SP		2.20 (0.12)***		0.92 (0.17)***
TB to SP		1.98 (0.08)***		0.82 (0.10)***
SP to IN		−1.45 (1.00)		−1.08 (1.33)
IN to IN		3.33 (0.28)***		2.31 (0.93)*
TA to IN		1.19 (0.52)*		1.43 (1.16)
TB to IN		0.58 (1.01)		0.14 (1.74)
SP to TA		−0.81 (0.45)		0.09 (0.68)
IN to TA		2.95 (0.25)***		2.22 (0.78)**
TA to TA		1.26 (0.37)***		1.24 (0.83)
TB to TA		2.26 (0.32)***		2.16 (0.69)**
SP to TB		−1.03 (0.26)***		0.73 (0.44)
IN to TB		3.08 (0.29)***		3.32 (0.71)***
TA to TB		2.72 (0.24)***		3.22 (0.55)***
TB to TB		1.92 (0.22)***		2.49 (0.59)***
*Structural terms*				
Asymmetric edges			NS	1.31 (0.40)**
GWID			10.18 (0.30)***	10.44 (0.35)***
GWDSP			−2.72 (0.07)***	−2.52 (0.09)***
GWESP			4.59 (0.29)***	2.28 (0.36)***
*Fit*				
Akaike information criteria	20,372	14,940	17,394	12,397

^a^*** <0.001; ** <0.01; * <0.05.

*^b^E, east; NW, north-west; S, south; SW, south-west*.

*^c^SP, small producer; IN, industrial; TA, type A; TB, type B*.

**Table 6:** **Parameter coefficients and fit for the four exponential random graph models (ERGMs) of pig trade in an extensive production system (Spain—Extremadura)**.

Covariates	ERGM coefficients (SE)^a^			
	Bernoulli (edges)	Edges + attributes	Edges + network statistics	Edges + attributes + network statistics
*Edges*	−10.77 (0.02)***	−9.34 (0.04)***	−5.87 (0.26)***	−4.87 (0.55)***
*Nodal attribute mixing*				
Housing system^b^				
In to in		−1.23 (0.05)***		−0.74 (0.06)***
In to out		−0.68 (0.04)***		−0.30 (0.05)***
Out to in		−0.68 (0.04)***		−0.56 (0.05)***
Out to out		Reference		Reference
Type of farm^c^				
MU to MU		2.44 (1.00)*		2.09 (2.47)
MU to FA		1.79 (0.58)**		1.84 (0.88)*
MU to FF		−0.26 (0.27)		−0.34 (0.28)
MU to FI		−0.23 (0.38)		−0.41 (0.46)
MU to SP		NA		NA
FA to MU		1.38 (0.71)		0.83 (1.17)
FA to FA		0.07 (0.58)		−0.09 (0.82)
FA to FF		−0.39 (0.13)**		−0.45 (0.16)**
FA to FI		0.18 (0.13)		0.06 (0.18)
FA to SP		−0.89 (0.71)		−0.67 (0.87)
FF to MU		0.20 (0.22)		0.36 (0.32)
FF to FA		−0.67 (0.14)***		−0.28 (0.18)
FF to FF		−1.25 (0.05)***		−0.75 (0.06)***
FF to FI		−0.72 (0.05)***		−0.33 (0.06)***
FF to SP		−2.46 (0.26)***		−1.83 (0.28)***
FI to MU		0.47 (0.27)		0.28 (0.36)
FI to FA		−0.02 (0.15)		−0.05 (0.21)
FI to FF		−0.59 (0.05)***		−0.50 (0.06)***
FI to FI		Reference		Reference
FI to SP		−1.79 (0.27)***		−1.52 (0.31)***
SP to MU		0.15 (1.00)		0.96 (1.27)
SP to FA		NA		NA
SP to FF		−2.46 (0.26)***		−1.37 (0.29)***
SP to FI		−1.95 (0.29)***		−1.00 (0.30)***
SP to SP		−2.12 (1.00)*		−0.86 (1.39)
*Structural terms*				
Isolates			1.08 (0.04)***	0.90 (0.06)***
Asymmetric edges			−1.99 (0.27)***	−2.28 (0.54)***
GWOD			−2.59 (0.06)***	−2.55 (0.08)***
GWDSP			−0.24 (0.02)***	−0.26 (0.03)***
GWESP			3.63 (0.27)***	4.34 (0.24)***
*Fit*				
Akaike information criteria	96,949	94,851	91,182	89,952

^a^*** <0.001; ** <0.01; * <0.05.

*^b^In, indoor; Out, outdoor*.

*^c^U, multipliers; FA, farrow farms; FF, farrow-to-finish farms; FI, finishers; SP, small producers*.

**Table 7:** **Parameter coefficients and fit for the four exponential random graph models (ERGMs) of pig trade in an intensive production system (France—Côtes-d’Armor)**.

Covariates	ERGM coefficients (SE)^a^			
	Bernoulli (edges)	Edges + attributes	Edges + network statistics	Edges + attributes + network statistics
*Edges*	−7.52 (0.02)***	−11.08 (0.28)***	−4.27 (0.15)***	−6.44 (0.46)***
*Homophily*				
Housing system		1.11 (0.24)***		0.72 (0.30)*
*Nodal attribute mixing*				
Pig company^b^				
No. 1 to no. 1		1.56 (0.15)***		1.07 (0.21)***
No. 1 to no. 2		−2.23 (0.72)**		−2.69 (0.88)**
No. 2 to no. 2		2.93 (0.21)***		2.78 (0.30)***
NC to NC		Reference		Reference
Type of farm^c^				
MU to MU		3.72 (0.27)***		1.53 (0.77)***
FA to MU		0.98 (1.01)		−1.06 (2.93)
FF to MU		−1.10 (0.71)		−2.69 (1.65)
FI to MU		−0.57 (0.59)		−0.21 (1.24)
SP to MU		NA		NA
TR to MU		NA		NA
MU to FA		4.21 (0.22)***		2.81 (0.37)***
FA to FA		0.92 (1.00)		−0.12 (1.25)
FF to FA		−0.14 (0.42)		−1.09 (0.74)
FI to FA		−1.12 (0.71)		−2.03 (1.61)
SP to FA		NA		NA
TR to FA		NA		NA
MU to FF		4.64 (0.11)***		3.08 (1.69)***
FA to FF		2.07 (0.17)***		0.79 (0.26)**
FF to FF		0.90 (0.12)***		−0.11 (0.17)
FI to FF		−1.28 (0.22)***		−1.50 (0.35)***
SP to FF		NA		NA
TR to FF		2.58 (0.76)***		5.45 (0.27)***
MU to FI		2.17 (0.17)***		0.53 (0.23)*
FA to FI		3.40 (0.12)***		2.07 (0.17)***
FF to FI		2.57 (0.10)***		1.66 (0.13)***
FI to FI		Reference		Reference
SP to FI		NA		NA
TR to FI		3.12 (0.51)***		7.99 (0.26)***
MU to SP		3.30 (1.01)**		1.72 (1.41)
FA to SP		NA		NA
FF to SP		NA		NA
FI to SP		0.29 (1.00)		0.41 (1.42)
SP to SP		5.28 (1.02)***		4.25 (2.12)*
TR to SP		NA		NA
MU to TR		7.13 (0.30)***		4.65 (0.33)***
FA to TR		6.69 (0.32)***		5.54 (0.28)***
FF to TR		7.05 (0.12)***		5.91 (0.10)***
FI to TR		3.81 (0.31)***		3.41 (0.39)***
SP to TR		NA		NA
TR to TR		NA		NA
*Structural terms*				
Isolates			0.94 (0.08)***	0.28 (0.10)**
Asymmetric edges			−1.53 (0.15)***	−2.42 (0.29)***
GWOD			−2.76 (0.07)***	−1.77 (0.13)***
GWDSP			−0.27 (0.01)***	−0.18 (0.04)***
GWESP			2.51 (0.10)***	0.85 (0.17)***
*Fit*				
Akaike information criteria	53,087	40,233	48,891	39,649

^a^*** <0.001; ** <0.01; * <0.05.

*^b^For readability, not all values for selective for the pig companies are shown; NC, no company*.

*^c^MU, multipliers; FA, farrow farms; FF, farrow-to-finish farms; FI, finishers; SP, small producers; TR, trade operators*.

## Discussion

Exponential random graph models were used to represent, understand, and predict pig trade networks structures from different European production systems, with predominantly small-scale, extensive, or intensive pig producers. Such information improved our understanding of the processes that govern the organization of pig trade and can further be used to better inform European policy makers on prevention and control measures against swine diseases such as African swine fever, classical swine fever, or porcine reproductive and respiratory syndrome.

Rapidity of targeted action during the initial phase of an outbreak is fundamental to effectively curtail the transmission and minimize the disease burden. At this time, movements of animals have not been banned, and it is thus relevant to use “peace-time” movement networks to compare different control strategies. Until recently, variability in contact patterns was mostly approached in epidemic models by combining probabilities of contact between premises according to their type of production and to the distance between premises (22, 23). These efforts may fail to capture the structural properties of livestock trade networks that will impact diseases dynamic as well as their spatial spread (24–26). The use of ERGMs to model pig trade networks allows capturing both network topology and complex behaviors that depend on various premises characteristics. They may thus help to generate more realistic networks that may be used to study diseases spread, identify premises that could be targeted for risk-based surveillance, early detection, and rapid control of diseases, and compare different control strategies (27–29). Indeed, by simulating diseases spread on several simulated networks, we could identify some farms that are frequently and early infected and thus that should be targeted to provide timely and accurate indications of epidemic activity (30, 31). These networks could also be simulated to assess the effectiveness of compartmentalization or zoning, strategies that might be efficient to prevent disease’s spread without disrupting pig trade (32, 33).

Exponential random graph models developed in this study also improved our understanding of the drivers of pig trade in different production systems. Geographic mixing patterns strongly structured pig trade organization in the small-scale production system, whereas belonging to the same company, or keeping pigs in the same housing system appeared to be key drivers of pig trade, in intensive and extensive production systems, respectively. As expected, the specialization and organization of pig production also explained a part of trading behaviors, illustrated by the heterogeneous mixing between types of production. This mechanism was however less important than the geographical location of premises and as important as belonging to the same pig company in the small-scale and intensive production systems, respectively. Geographical proximity did not appear to play a role in the intensive system, whereas it was significant in the small-scale producer system. Unfortunately, model degeneracy occurred when trying to include the distance between premises as a covariate in the extensive production system (Spain—Extremadura), preventing a conclusion to be drawn on the impact of this covariate in this production system.

Finally, this study revealed that the inclusion of nodal attributes was necessary to represent the mixing patterns, but it was not sufficient to reproduce the great clustering observed in the pig trade networks, which could only be represented when adding additional statistics on local network configurations. These statistics reveal some features of these networks, such as the propensity of trade to have a short path length (negative coefficient for the degree distribution terms). Some social behavior, economic factors, or unobserved covariates, which may differ between countries, may also have driven the choices of farmers for trading partners (e.g., pig prices, road distribution, traditions, or cultural practices, etc.). This may explain the increased clustering as represented by the positive coefficient for the *gwesp* term.

Until recently, problems of degeneracy and computational intractability for large network sizes limited the use of ERGMs in epidemiological modeling (11, 34). Indeed, ERGMs have been mainly used on small networks to understand the factors driving human social behaviors (35, 36) and have sometimes been applied on disease transmission modeling (37). Ortiz-Pelaez et al. (38) were the first to introduce ERGMs in preventive veterinary medicine, using this method to understand the factors driving livestock trade in a small network of villages in Ethiopia. In the present study, the use of new parameters that limit degeneracy problems (12) allowed us to obtain statistical models with a good fit to the large-size observed networks. Isolates always depends on the spatial and temporal “frontiers” that are decided and on the exchange with farms outside these “frontiers.” For Extremadura, most of movements were inside the region (i.e., from the 9,544 isolates, 174 of them sent pigs to farms outside Extremadura and 73 received pigs from farms outside Extremadura); therefore, most of the isolates (97.4%) can be considered as true isolates during the study period (i.e., not movements within the region and not trading with other regions). For Côtes-d’Armor, there was a lot of exchange with other departments, and only 47% of the 489 isolates can be considered as true isolates (i.e., 57 sent pigs to farms outside Côtes-d’Armor and 202 received pigs from farms outside the region). Therefore, the scale to simulate networks for disease spread models should consider areas where almost all movements are inside these areas. For Bulgaria, the entire country was evaluated, and all were considered to be true isolates; however, it was not feasible to include these isolates in the ERGM due to memory limits, and therefore the model produced here might not fully represent the true pig movement network in Bulgaria. Further studies should be conducted to validate this network once computational difficulties to fit ERGM’s to large networks are solved.

Since implementation of Regulation (EC) no 1760/2000 of the European parliament, recording of livestock movements between premises is mandatory, making data on pig trade movements available, at least in the main producing countries in the EU. However, there are no standards on the definition of different types of premises (e.g., backyard) or on other premises attributes. The scales of the networks considered in this study were also different, being the national level for Bulgaria and the regional level for France and Spain, to better study very specific production systems. Therefore, though the analyses and results in the different settings intended to illustrate the applicability and usefulness of the approach in the predominant swine production systems in the EU, mechanisms and rules that govern trade organization in the different study populations are not fully comparable.

Several studies showed that, in addition to the topology of a contact network, heterogeneity in the weight of edges and temporal network dynamics had a strong influence on diseases spreading (39, 40). Tools to model such networks are still under development, and their application is currently limited by the size of the networks modeled (41–43). In the next few years, these methods could be promising tools to improve our representation of real-world livestock trade networks.

## Conclusion

This study is one of the very first to illustrate the usefulness of ERGMs to understand and simulate livestock trade networks under different European production systems, specifically small-scale, extensive, and intensive swine production systems. Depending on the production system, some premises characteristics, such as their geographical location, type of production, belonging to a pig company or housing system, were key drivers of pig trade, but adding statistics on local network configurations was necessary to accurately capture the great clustering observed in all pig trade networks. These models offer a framework to simulate realistic pig trade networks that may be included in epidemic models to compare different control strategies against major swine diseases such as African swine fever, classical swine fever, or porcine reproductive and respiratory syndrome.

## Author Contributions

AR, BM-L, and VG designed the study and developed the R codes. AR and BM-L gathered, cleaned, and verified the data. TA, AW-S, SM, EE, and JS-V contributed to the interpretation and critical discussion of the nature, characteristics, and structure of the data for the different study regions. AR carried out the analyses and wrote the draft of the manuscript. All authors participated in the interpretation and discussion of the results, read, edit, and approved the final manuscript.

## Conflict of Interest Statement

The authors declare that the research was conducted in the absence of any commercial or financial relationships that could be construed as a potential conflict of interest. The reviewer AB and handling Editor declared their shared affiliation, and the handling Editor states that the process nevertheless met the standards of a fair and objective review.
